# Impaired response of memory Treg to high density lipoproteins is associated with intermediate/high cardiovascular disease risk in persons with HIV

**DOI:** 10.3389/fimmu.2023.1146624

**Published:** 2023-03-10

**Authors:** Laura Atehortua, Mirza Baig, Jamie Morris, Sarah Trentman, W. Sean Davidson, Carl J. Fichtenbaum, Claire A. Chougnet

**Affiliations:** ^1^ Division of Immunobiology, Cincinnati Children’s Hospital Research Foundation, University of Cincinnati College of Medicine, Cincinnati, OH, United States; ^2^ Division of Infectious Diseases, Department of Internal Medicine, University of Cincinnati College of Medicine, Cincinnati, OH, United States; ^3^ Division of Experimental Pathology, Department of Pathology and Laboratory Medicine, University of Cincinnati, Cincinnati, OH, United States

**Keywords:** HIV, cardiovascular disease, regulatory T cells, HDL, oxidative stress, inflammation

## Abstract

Cardiovascular disease (CVD) is a leading cause of enhanced morbidity and mortality in persons with HIV (PWH) in the era of highly active antiretroviral therapy (AART). However, the underlying mechanisms are not fully understood. Regulatory T cells (Treg), notably the highly suppressive memory subset, have been shown to limit CVD. Importantly, memory Treg cell numbers remain low in many treated PWH. High density lipoproteins (HDL) also protect from CVD, and we previously found that Treg-HDL interactions reduce oxidative stress in these cells. Here, we evaluated Treg-HDL interactions in PWH and whether they were operative in those higher CVD risk. To do that, we recruited a cohort of PWH with intermediate/high CVD risk (median ASCVD risk score of 13.2%, n=15) or low/borderline risk (median ASCVD risk score of 3.6%, n=14), as well as a group of statins treated PWH with intermediate/high CVD risk (median ASCVD risk score of 12.7%, n=14). We evaluated Treg frequency, phenotype and response to HDL. PWH with Int/High CVD risk had a significantly lower number of memory Treg, but memory Treg were more activated and displayed an inflammatory phenotype, versus those with Low/BL CVD risk. In untreated patients, Treg absolute numbers were negatively correlated with ASCVD score. Although HDL decreased oxidative stress in memory Treg in all subjects, memory Treg from PWH with Int/High CVD risk were significantly less responsive to HDL than those from PWH with Low/BL CVD risk. The level of oxidative stress in memory Treg positively correlated with ASCVD scores. In contrast, plasma HDL from PWH, regardless of CVD risk, retained their anti-oxidative properties, suggesting that the defect in memory Treg response to HDL is intrinsic. Statin treatment partially ameliorated the memory Treg defect. In conclusion, the defective HDL-Treg interactions may contribute to the inflammation-induced increased CVD risk observed in many AART-treated PWH.

## Background

Persons with HIV (PWH) exhibit increased risk of cardiovascular diseases (CVD), including acute myocardial infarction, stroke, peripheral artery disease, heart failure and sudden cardiac death (1). HIV-associated CVD have been related with persistent chronic inflammation (2, 3), characterized by high levels of T cell activation and increased levels of circulating pro-inflammatory cytokines (3).

Regulatory T cells (Treg) are a specialized subset of CD4+ T cells that are critically to dampening inflammation (4, 5). Treg are involved in the protection against CVD (6, 7). Indeed, partial depletion of Treg increases plaque formation and inflammation in Apoe^-/-^ mice (8). In addition, the depletion of Treg in a LDLR^−/−^ mouse model, resulted in increased plasma cholesterol and VLDL levels (9). Treg with a lesser ability to suppress the inflammatory response can also infiltrate the heart and reduce heart function by inducing cardiac inflammation (10). Chronic HIV infection affects the numbers and activity of Treg, notably memory Treg, the most suppressive and activated subset. Despite active antiretroviral therapy (AART) suppression, absolute numbers of memory Treg remain low in PWH, while naïve Treg numbers are not changed (11, 12). As pathways supporting memory and naïve Treg survival are distinct, memory Treg being notably less dependent on IL-2 than naïve Treg (13, 14), these data suggest that some pathways involved in memory Treg homeostasis are not fully operational in PWH, despite antiretroviral suppression of viral replication. Such defects may thus play a role in PWH increased CVD risk.

Mitochondrial reactive oxygen species (mtROS) are critical for Treg function and differentiation (15). Oxidative stress arises from an imbalance in the production and scavenging rates of ROS. Increased mitochondrial oxidative stress and cell death in Treg have been observed in CVD (16, 17). Moreover, chronic inflammation in HIV can lead to an increased production of ROS (18). Oxidative stress has been mainly described to affect CD4+ T cells, monocytes, dendritic cells and neutrophils in PWH (19, 20). The exact mechanisms driving the increased ROS in PWH, and whether it is linked to Treg homeostasis, are not well understood.

We previously demonstrated (21) a novel connection between lipoprotein metabolism and Treg homeostasis. We observed that high density lipoproteins (HDL) promotes memory Treg survival and enhanced mitochondrial metabolism (21). More recently, we found that memory Treg exhibit increased mitochondrial oxidative stress compared to naïve Treg, and HDL blunt the oxidative stress promoting memory Treg homeostasis. Here, we investigate the frequency and phenotype of Treg subsets in PWH at higher and lower risk for CVD. We also studied the interactions between HDL and memory Treg in the same groups of patients.

## Methods

### Patient recruitment and sample collection

A total of 43 ART-suppressed PWH were recruited based upon their estimated CVD risk and matched for age (+/- 10 years), sex, and smoking status (see **Table 1** for demographics). Atherosclerotic Cardiovascular Disease (ASCVD) pooled risk equation score was estimated, taking in consideration age, sex, race, cholesterol levels, blood pressure, medication use, diabetic status, and smoking status as described (22). Based on guidelines from the American Heart Association and American College of Cardiology (23, 24), including in PWH (25), we considered patients to have a low/borderline (Low/BL) CVD risk if their ASCVD was ¾ 7.5% whereas an ASCVD score > 7.5% was considered as an intermediate/high CVD risk (Int/High). In addition, a group of statin-treated PWH were recruited (n=14). These had been prescribed statins for a median of 231 weeks (range: 28-535 weeks) prior to obtaining blood samples for this study. The statins utilized were atorvastatin (N=6), rosuvastatin (N=6) and one each on pravastatin and simvastatin. Fasting heparinized blood samples were obtained for analysis of plasma and cells. Blood samples from healthy HIV-uninfected donors (HC) were obtained from Hoxworth Blood Center (Cincinnati, OH).

**Table 1:** Characteristics and demographics of study participants. Results are shown as median (range).

Parameter	Low/BL CVD Risk (N=14)	Int/High CVD Risk (N=15)	Statin (N=14)
**Median Age (yrs)**	50	61	60
Sex			
**Male**	73%	80%	87%
**Female**	27%	20%	13%
**Median (range)** **CD4 count** **(cells/mm^3^)**	676 (1779 – 243)	588 (1077 – 151)	623 (1377 - 200)
**HIV viral load (copies/mL)**	<20	<95	<28
**Median (range) ASCVD PCE**	3.6 (0.6 – 6.6) %	13.2 (8.6 – 25.6)%	12.7 (6.0 – 32.4)%
**Total Cholesterol (range) (mg/dL)**	193 (293 – 113)	187 (274 – 144)	186 (235 - 109)
**HDL (range) (mg/dL)**	49 (88 – 35)	48 (64 – 35)	49 (86 - 25)
**LDL (range) (mg/dL)**	123 (187 – 54)	115 (165 – 62)	105 (145 – 27)

All comparisons between the groups were non-significant (p>0.05 ANOVA test). ASCVD, Atherosclerotic cardiovascular disease pooled risk equation score.

### Cell and plasma isolation

Fasting heparinized peripheral blood was centrifuged at 2000 rpm, 10 min, 22°C to collect the plasma. Aliquots of plasma were frozen at -80°C. The remaining blood was diluted with PBS 1:2. Peripheral blood mononuclear cells (PBMC) were separated by centrifugation through Ficoll-Hypaque (GE, Fairfield, CT). Aliquots of PBMC were frozen in FBS + 10% DMSO and stored in liquid nitrogen. CD4+ T cells were purified from the cryopreserved PBMC by negative selection using the Miltenyi CD4 negative separation kit (Auburn, CA), per the manufacturer’s instructions. In some experiments, healthy control Treg were used. To isolate bulk Treg, CD4+ T cells were stained with live dead aqua (Thermofisher, Waltham, MA), anti-CD8-FITC, anti-CD25-APC (BD Pharmingen, San Diego, CA), anti-CD127-PE (Beckman Coulter, Fullerton, CA), and sorted using a FACS Aria (BD). Purity of the sorted Treg was >90%, as determined by post-sorting analysis of FOXP3 expression.

### Cytokine production

1x10^6^ PBMC from PWH were activated with PMA (50 ng/ml), ionomycin (1µg/ml) and brefeldin A (10 µg/ml) (Sigma-Aldrich) for 4h at 37°C in RPMI 1640 media containing 10% FBS (Thermofisher). Cells were washed with PBS (Thermofisher) and stained for flow cytometry analysis.

### Flow cytometry analyses of blood subsets

PBMC or CD4+ T cells from PWH were thawed and stained with live dead blue (Thermofisher) for 15 min at RT. Cells were washed with PBS 2% FBS, resuspended in blocking buffer (PBS 2% FBS 20 µg/ml human IgG) and stained with the surface antibodies: CD45RA, CD95, CD25, CD39, HLA-DR, CD69, CD3, CD4, and CD127 (see **Table S1**). 10 µl of brilliant stain buffer (BD) was added to each sample. Cells were incubated for 30 min at RT and washed with PBS 2% FBS. Cells were fixed and permeablized with FOXP3 perm/fix buffer kit (Thermofisher) for 1h at 4°C, followed by intracellular staining with FOXP3, ICOS, Ki67, CTLA-4, TNF-α and IFN-γ (see **Table S1**) for 30 min at 4°C. Samples were acquired on the Aurora flow cytometer (Cytek, Bioscience, CA). PBMC from two healthy controls (HC) were included in every run to check for possible batch effect. Flow cytometry data were analyzed using the Flowjo software v10.6.2.

### HDL isolation

HDL were isolated by density ultracentrifugation, as described in one of our studies (26). Briefly, density of plasma was adjust to 1.063 g/ml with KBr in Optiseal tubes (Beckman Coulter, Brea, CA) and centrifuged for 18 h at 40,000 RPM 199,800 RCF at 15°C, using the rotor 50.4 Ti (serial # O2U 879, Beckman), to float LDL and VLDL. These were discarded and the density was adjusted to 1.21 g/ml with KBr to float total HDL at 40,000 rpm for 18 h at 15°C (27). HDL were collected at the top of the tube and the KBr was removed by desalting columns (Thermofisher). Protein concentrations were determined by the modified Markwell-Lowry assay (28). SDS-PAGE with Coomassie staining was performed on the HDL to confirm purity (via absence of significant APOB and plasma albumin) and particle size was ascertained by native PAGE (**Figure S1**). In some experiments, a commercially available pool of HDL from healthy controls (Sigma-Aldrich, St. Louis, MO) was utilized.

### Treg response to HDL

Purified CD4+ T cells from PWH or purified Treg from HC were cultured in the serum-free medium, X-VIVO 15 (Lonza, Charleston, TN) overnight at 37°C in the presence or absence of control HDL (700 µg/ml; Sigma-Aldrich) or different HDL preparations from PWH (700 µg/ml) as described in the legends. ROS expression was quantified using MitoSOX Red probe (500 mM, Thermofisher), for 30 min at 37°C, before surface staining. Cells were washed with PBS 2% FBS and stained with the surface antibodies: CD4, CD127, CD25, CD45RA, CD95 and HLA-DR (see **Table S1**). Samples were acquired on the spectral cytometer Aurora (Cytek). Flow cytometry data were analyzed using the Flowjo software v10.6.2 (BD).

### Statistics

Graphics and Statistical analyses were generated with GraphPad Prism software, version 9.3.1 (GraphPad). Most analyses, when the Treg phenotype was compared between the different PWH groups, used unpaired t-tests. T-test was also applied to compare the effect of HDL in the percentage of Mitosox-expressing memory Treg from PWH. ANOVA one-way tests were used to compare Treg cytokine production between the different groups of PWH. Pearson correlation was utilized to determine the association between Treg absolute numbers, their response to HDL and ASCVD score. Mean and standard deviation are used as representation of the data. For all statistics a p-value ¾ 0.05 was considered significant.

## Results

### Characteristics of study populations

The demographic and clinical characteristics of the study populations are shown in **Table 1**. As described in the Material and Methods, three groups were recruited based on their ASCVD score (low/BL CVD risk, Int/High CVD risk and statin group). All participants had a HIV viral load < 95 copies/ml. Plasma levels of total cholesterol, HDL cholesterol and LDL cholesterol were similar between the three groups (p>0.05, ANOVA tests, see **Table 1**).

### PWH with Int/High CVD risk have decreased absolute numbers of total and circulating memory Treg

**Figure 1:**
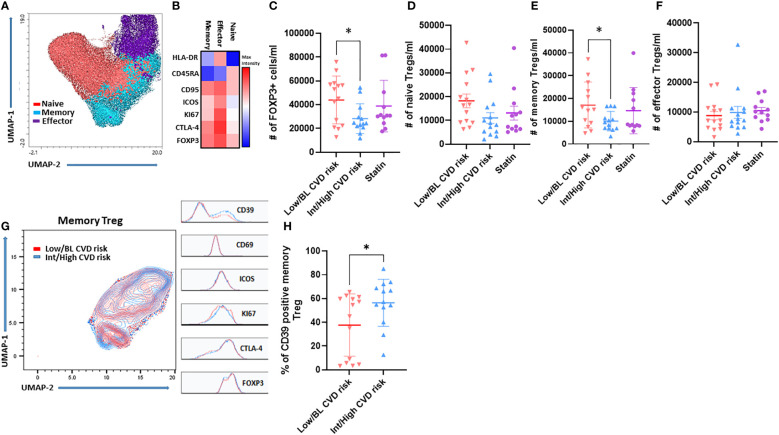
Memory Treg frequency decreases in PWH with Int/High CVD risk but they are more activated. PBMC were isolated from PWH and analyzed by flow cytometry. **(A)** UMAP and **(B)** FlowSOM clustering of Treg subsets. Absolute number of **(C)** Total Treg **(D)** Naïve, **(E)** Memory and **(F)** Effector Treg. **(G)** UMAP of memory Treg from PWH with Int/High (blue) and Low/BL CVD risk (red). **(H)** Percentage of CD39 memory Treg from PWH with Int/High and Low/BL CVD risk. Asterisk indicate significant differences (*p<0.05) in t-test.

Circulating Treg subsets were analyzed in the 43 participants by flow cytometry. The unbiased clustering by UMAP and FlowSOM identified three main subsets (**Figure 1A**) after gating in the CD3+CD4+CD25+FOXP3+ cells (see **Figure S2A** for gating strategy). According with the expression of CD45RA, CD95, HLA-DR, ICOS, CTLA-4, Ki67 and FOXP3, these were classified as naïve, memory and effector Treg (**Figure 1B**). Naïve and memory Treg were the most abundant in circulation compared with effector Treg (**Figure S2B**). Importantly, PWH with Int/High CVD risk had significant lower absolute numbers of total Treg (FOXP3+) (**Figure 1C**) and memory Treg (CD4+FOXP3+CD25+CD95+) than PWH with Low/BL CVD risk (**Figure 1E**). Statin-treated PWH exhibited an intermediate number of memory Treg compared with PWH with Int/High and Low/BL CVD risk (**Figure 1E**). In addition, absolute numbers of total Treg and memory Treg inversely correlated with ASCVD in the untreated groups combined (**Figures S3A, B**), further suggesting that the deficit in Treg, particularly memory Treg, is more pronounced in persons with higher CVD risk. In contrast, there was no significant change in the absolute number of naïve and effector Treg between the three groups of PWH (**Figures 1D, F** **)**. Next, we analyzed the expression of the activation markers CD39, CD69, ICOS, KI67, CTLA-4 and FOXP3 in memory Treg, restricting our analysis to PWH with Int/High CVD risk and Low/BL CVD risk (**Figure 1G**). PWH with Int/High CVD risk had a higher percentage of CD39+ memory Treg (**Figure 1H**), but similar expression of the other activation markers.

### Memory Treg from PWH with Int/High CVD risk exhibit an exacerbated pro-inflammatory profile

**Figure 2:**
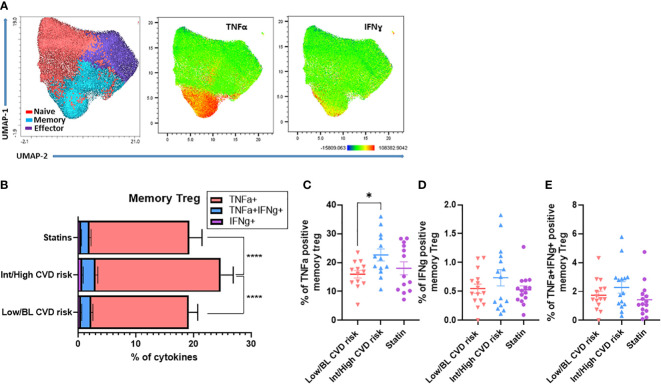
Memory Treg from PWH with Int/High CVD risk have a higher production of pro-inflammatory cytokines. PBMC from PWH were treated with PMA (50 ng/ml), ionomycin (1 µg/ml) and brefeldin A (10 µg/ml) for 4h at 37°C. Production of TNF-α and IFN-γ in Treg subsets was analyzed by flow cytometry. **(A)** UMAP of Treg subsets producing TNF-α or IFN-γ. **(B)** Production of TNF-α, IFN-γ and TNF-α/IFN-γ in memory Treg from PWH with Int/High CVD risk, Low/BL CVD risk and PWH using statins. ANOVA one way indicates significant differences (****p<0.0001) between the three groups. Percentage of memory Treg producing **(C)** TNF-α, **(D)** IFN-γ and **(E)** TNF-α/IFN-γ. Asterisk indicate significant differences (*p<0.05) in t-test.

Next, circulating Treg were stimulated with PMA/ionomycin to evaluate their capacity to produce pro-inflammatory cytokines (TNF-α and IFN-γ). The unbiased clustering by UMAP showed memory Treg from PWH are the main producers of TNF-α and IFN-γ, compared with naïve and effector Treg (**Figure 2A**). When the polyfunctionality of memory Treg was compared between the 3 patient groups, we found that memory Treg from PWH with Int/High CVD risk produced the highest levels of TNF-α or IFN-γ, alone or together (**Figure 2B**). TNF-α production was significantly increased in memory Treg from PWH with Int/High CVD compared with those with Low/BL CVD risk (**Figure 2C**). Interestingly, individuals on statins exhibited decreased levels of pro-inflammatory cytokines like those of PWH with Low/BL CVD risk (**Figures 2B–E**). These data suggest memory Treg from PWH with Int/High CVD risk have a pro-inflammatory phenotype that may contribute to the persistent chronic inflammation observed in those patients.

### High density lipoproteins are less efficient at reducing mitochondrial oxidative stress in memory Treg from PWH with Int/High CVD risk

**Figure 3:**
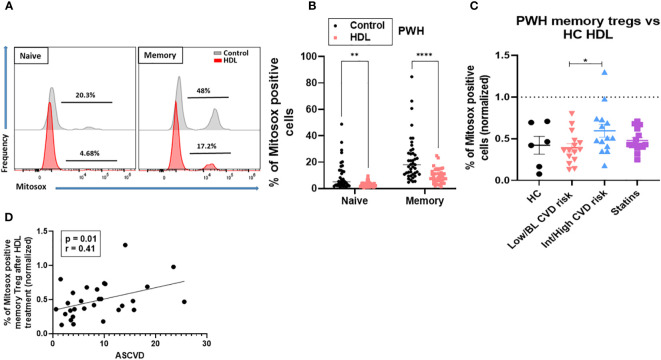
HDL-Treg interactions are defective in blunting ROS production in memory Treg from PWH with Int/High CVD risk. mtROS levels were measured in naïve and memory Treg cultured with or without HDL (700 µg/ml) in X-VIVO medium overnight, using the MitoSOX Red probe (500 mM). **(A, B)** Percentage of Mitosox positive cells was estimated in 43 PWH from the three groups. Asterisks indicate significant differences (**p<0.005, ****p<0.0001) in 2-way ANOVA test. **(C)** Percentage of ROS production was normalized versus the level of Mitosox expression in cells without HDL treatment (control, dotted line) in HC (n= 6), PWH with Low/BL (n=15) or Int/High CVD risk (n=15) and under statins (n=14). Asterisks indicate significant differences (* p<0.05) in t-test. **(D)** Pearson correlation between ASCVD and percentage of Mitosox positive memory Treg after HDL treatment. P and r values are displayed in the figure (* p<0.05 for significant differences).

Oxidative stress is a key factor in the pathophysiology of CVD. We investigated whether HDL have anti-oxidative properties in Treg subsets. First, we evaluated the capacity of HDL to blunt mitochondrial oxidative stress in memory Treg from PWH. Total CD4 T cells from PWH were exposed to pooled HDL from healthy controls (HC). Memory Treg from PWH expressed more mitochondrial ROS (mtROS) than naïve Treg, as evidenced by higher percentage of MitoSOX Red (O2•- sensitive mitochondrial probe) cells. mtROS levels were significantly decreased by culture with HDL overnight (**Figures 3A, B**). We focused on memory Treg because they were the only subset altered in absolute number in PWH with Int/High CVD risk (**Figure 1E**) and used naïve Treg as a negative control, because their response to HDL was minimal (**Figures 3A, B** **)**. When the response to HDL was normalized versus the level of Mitosox expression in cells without HDL treatment (**Figure 3C** control, dotted line), memory Treg from PWH with Int/High CVD risk were significantly less responsive to HDL compared to those from HC or PWH with Low/BL CVD risk. In addition, the level of Mitosox expression by memory Treg after HDL treatment positively correlated with ASCVD score, indicating that Treg from PWH with Int/High CVD risk exhibit a higher mitochondrial oxidative stress, even in presence of HDL (**Figure 3D**). PWH on statins displayed an intermediate response to HDL between PWH with Low/BL or Int/High CVD risk (**Figure 3C**). This data suggests there is an intrinsic impairment of memory Treg in PWH with Int/High CVD risk, which is not fully corrected by statins or HDL.

### HDL from PWH, regardless of CVD risk, was fully capable of controlling oxidative metabolism in memory Treg from HC

**Figure 4:**
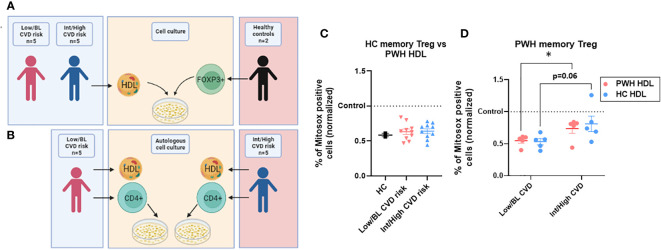
HDL from PWH with Int/High CVD risk are as functional as those from Low/BL CVD risk or HC. **(A)** HDL was isolated from plasma of 5 PWH with Int/High CVD risk and 5 with Low/BL CVD risk. Treg were sorted from 2 HC and tested against the 10 samples of HDL for 24h in X-vivo media. **(B)** Autologous HDL (700 µg/ml) was given to autologous CD4 T cells from PWH with Int/High CVD risk (n=5) and Low/BL CVD risk (n=5) for 24h in X-vivo media. **(C)** Percentage of ROS production in memory Treg was evaluated and the data were normalized versus the level of Mitosox expression in cells without HDL treatment (control, dotted line). Non-significant differences in t-test. **(D)** Percentage of ROS production was normalized in memory Treg versus the level of Mitosox expression in cells without HDL treatment (control, dotted line) in PWH with Low/BL or Int/High CVD risk. Asterisks indicate significant differences (* p<0.05) in t-test.

Next, we determined whether the HDL from PWH with Int/High CVD were also defective. We isolated the HDL from the plasma of 10 PWH with Int/High or Low/BL CVD (5 in each group) (**Figure S1**) and tested their anti-oxidative properties against Treg from 2 HC. The experimental scheme is depicted in **Figure 4A**. We chose individuals with the highest memory Treg absolute numbers in the Low/BL CVD group, and those with the lowest memory Treg numbers for the Int/High CVD risk group (see **Figure 1E** and **Table S2**). PWH with Int/High CVD risk had lower HDL levels compared to the Low/BL CVD risk group (see **Table S2**). However, HDL from PWH, regardless of CVD risk, were able to blunt oxidative stress in memory Treg from HC when compared at an equal level (in terms of HDL protein). In fact, HDL from PWH performed as effectively as the HDL from HC (**Figures 4C, D** **)**. This indicates that the ‘quality’ of the HDL in PWH with high or low CVD risk is not defective with respect to Treg interactions. Then, we tested whether HDL from PWH with Int/High or Low/BL CVD risk were also defective at inducing an anti-oxidative response in their corresponding memory Treg (See **Figure 4B** for experimental scheme). Memory Treg from PWH with Int/High CVD risk were also significantly less responsive to their own HDL compared to those from PWH with Low/BL CVD risk (**Figure 4D**), similar to their low response to HDL from HC (**Figure 4D**).

## Discussion

Treg play an essential role in the modulation of inflammation and maintenance of immune homeostasis. The dysregulation in the generation and/or function of Treg appear to contribute to the development and progression of CVD. In this study, we investigated Treg subset frequency and phenotype in PWH with Int/High or Low/BL CVD risk. Because we had found that HDL interactions with memory Treg promote their survival, and limit their oxidative response, we also analyzed the interactions between memory Treg and HDL in PWH at Int/High or Low/BL risk for CVD. Herein we described new defects in memory Treg in PWH at Int/High CVD risk, affecting both their numbers and their phenotype. Importantly, we identified for the first time that memory Treg in PWH at Int/High CVD risk exhibited an intrinsically defective response to HDL. These data thus suggest that in these patients, the HDL anti-inflammatory function is impaired, which could contribute to the chronic inflammation in these PWH.

The identification of Treg subsets with different phenotypes and suppressive effects changes our understanding of Treg biology during HIV infection and CVD progression. One of our main findings is that memory Treg, and not naïve or effector Treg, are altered in number and phenotype in PWH with Int/High risk of CVD. Our data are in agreement with those from Simonetta and colleagues, who had reported decreased total Treg numbers in different contexts of HIV infection, and in AART-treated aviremic patients (12). Based on the expression of CD45RA, they had also found that memory Treg were preferentially affected during HIV infection (12). Our data extend this observation, as we found this defect to be particularly present in PWH with Int/High CVD risk. In addition, our data show that memory Treg from PWH with Int/High CVD risk exhibited an activated and pro-inflammatory phenotype with increased expression of CD39 and production of TNF-α. TNF-producing Treg have been also identified in other viral infections, such as hepatitis A virus. These inflammatory Treg showed reduced suppressive functions and were associated with severe liver injury (29). Together, these results suggest that both the reduced numbers of memory Treg, and the emergence of inflammatory memory Treg, may contribute to the persistent chronic inflammation associated with the increased CVD risk observed in many ART-treated PWH.

Importantly, our results point out towards an intrinsic defect in memory Treg homeostasis in PWH with Int/High CVD risk. We had previously reported that HDL, and no other types of cholesterol containing lipoprotein particles such as LDL or VLDL, can directly bind to the surface of Treg in HC, in a specific fashion as conventional CD4+ T cells did not bind HDL (21). These Treg–HDL interactions decreased apoptosis in Treg by promoting mitochondrial metabolism, suggesting that these two separate anti-inflammatory processes may work in concert to protect from CVD. In the present study, we also found that HDL blunt mitochondrial oxidative stress in memory Treg from HC and from PWH with Low/BL CVD risk but, importantly, this response was impaired in PWH with Int/High CVD risk. Enhanced oxidative stress in Treg, compared to conventional CD4+ T cells, had been reported in patients with chronic coronary syndrome (30), but to our knowledge, the fact that HDL control such detrimental oxidative stress is a new concept. Oxidative stress can have detrimental effects on T cells and induce apoptosis (31, 32). Mechanisms underlying such intrinsic defects remain unclear. We have reported that HDL is able to bind to the cell membrane of healthy control Treg (21), and defective binding could thus play a role. Alternatively, and non-exclusively, HDL may fail to alter the balance between pro- and antioxidant molecules in Treg from PWH with Int/High CVD risk. However, as the binding and signaling mechanism(s) have not been yet elucidated, this will require future studies. Nevertheless, our data strongly suggest that exacerbated oxidative stress may play a role in the reduction of the numbers of memory Treg in PWH with Int/High CVD risk, and that HDL fails to rescue them.

On the other hand, in this study we found HDL from PWH irrespective of their CVD risk remain functionally active, as HDL was capable to induce an anti-oxidative response in memory Treg from either HC or PWH. This was a somewhat unexpected result, as Theodoros and colleagues found a reduction of HDL anti-oxidant/anti-inflammatory properties for endothelial cells in HDL isolated from AART-treated PWH (33). Furthermore, during certain infections and chronic inflammation such as obesity, HDL composition and size also change, reducing particularly the content of certain apolipoproteins, such as APOA1 and APOE (34). Despite this, our data indicates that changes occurring in these patients do not affect the overall ‘quality’ of the HDL particles when it comes to their anti-oxidative effect. This suggests that whatever changes in HDL particle composition that occur along the course to increased CVD risk in these patients did not adversely impact these HDL particular functions, whereas it may affect other HDL properties. It is also to be noted that the patients we studied did not have significantly low HDL levels, so we cannot rule out that different results could have been seen in AART-treated PWH with dyslipidemia (35).

Statins have immunomodulatory actions during chronic inflammation, and they influence the number and the suppressive function of Treg. Our previous work showed statins significantly increased Treg frequency and FOXP3 mRNA levels at day 30 post-treatment in HC (36). However, in the current study, statin treatment did not fully correct the low frequency and pro-inflammatory profile of memory Treg, and their impaired response to HDL in PWH with Int/High CVD risk. AART-treated PWH under statins displayed intermediate levels of memory Treg, production of pro-inflammatory cytokines and intermediate response to HDL. These data are consistent with those from Rothan and colleagues, who also showed that statin treatment did not have a significant effect at improving Treg frequency (37). Statin treatment has also been associated with lower T cell activation and inflammation in AART-treated PWH. They found pravastatin was not as efficient as atorvastatin to decrease T cell activation and exhaustion (38). However, Treg were not analyzed in this study. It should be noted that in our study not all patients were on the same statin regimen, and the length of treatment was very variable (ranging from 28 weeks to 535 weeks), which may explain the fact that we did not see a clear effect of statins. Determining whether different statins have a differential effect on Treg profile and/or whether the length of treatment also plays an important role will require further studies done in controlled clinical trials that limit such variability.

In conclusion, we uncovered that memory Treg from PWH with Int/High CVD risk exhibit impaired anti-oxidative response to HDL. These defective HDL-Treg interactions may contribute to the reduced number of memory Treg we and others have reported in these patients. The failure of HDL cholesterol-raising agents to provide clinical benefits for CVD (39) has highlighted the need to better understand how HDL exert their pleiotropic effects on inflammation. Our study could thus open up a new metric to evaluate potential therapeutic interventions aimed at modulating Treg function and particularly at controlling oxidative stress in Treg from PWH with Int/high CVD risk.

## Data availability statement

The original contributions presented in the study are included in the article/**Supplementary Material**. Further inquiries can be directed to the corresponding author.

## Ethics statement

The studies involving human participants were reviewed and approved by University of Cincinnati IRB. The patients/participants provided their written informed consent to participate in this study.

## Author contributions

MB, ST, and CF: recruitment of patients. JM and WSD: methodology. LAC: methodology and data analysis. LAC and CC: writing—original draft preparation. LAC, CC, CF, and WSD: review and editing. All authors contributed to the article and approved the submitted version.
