# Supplementary material for: Impaired response of memory Treg to high density lipoproteins is associated with intermediate/high cardiovascular disease risk in persons with HIV
Source: Front Immunol. 2023 Mar 10;14:1146624. doi: 10.3389/fimmu.2023.1146624 (PMC10036595; doi:10.3389/fimmu.2023.1146624)
Supplement: Supplementary file 1 [file DataSheet_1.docx]

**SUPPLEMENTARY MATERIAL**

**Impaired response of memory Treg to high density lipoproteins is associated with high cardiovascular disease risk in persons with HIV**

Laura Atehortua^1^, Mirza Baig^2^, Jamie Morris^3^, Sarah Trentman^2^, W. Sean Davidson ^3^, Carl J. Fichtenbaum^2^ and Claire A. Chougnet^1*^

1. Division of Immunobiology, Cincinnati Children’s Hospital Research Foundation, University of Cincinnati College of Medicine, Cincinnati, OH.
2. Division of Infectious Diseases, Department of Internal Medicine, University of Cincinnati College of Medicine, Cincinnati, OH.
3. Division of Experimental Pathology, Department of Pathology and Laboratory Medicine, University of Cincinnati, Cincinnati, OH.

***Correspondence:**

Claire Chougnet: [Claire.Chougnet@cchmc.org](mailto:Claire.Chougnet@cchmc.org)

| **Antigen** | **Fluorochrome** | **Clone** | **Laser line** | **Company** |
| --- | --- | --- | --- | --- |
| CD45RA | BUV395 | HI100 |  | BD |
| L/D | Blue (450) |  | UV | Thermofisher |
| CD95 | BUV737 | DX2 |  | BD |
| FOXP3 | Efluor 450 | PCH101 |  | ebioscience |
| CD69 | BV480 | FN50 |  | BD |
| CD3 | BV510 | OKT3 | Violet | Biolegend |
| TNFa | BV605 | MAb11 |  | Biolegend |
| ICOS | BV711 | C398.4A |  | Biolegend |
| Ki67 | BV786 | B56 |  | BD |
| INFg | Alexa fluor 488 | 4S.B3 | Blue | ebioscience |
| CD4 | PerCp eFluor 710 | RPA T4 |  | Thermofisher |
| CD127 | PE | R34.34 |  | Beckman |
| CTLA-4 | PECy5 | BNI3 | Yellow green | BD |
| CD25 | PECy7 | 2A3 |  | BD |
| CD39 | APC | TU66 | Red | BD |
| HLA-DR | APC-Cy7 | L243 |  | BD |

**Table S1. Flow cytometry panel to identify Treg subsets**

| **HIV donor** | **Age** | **Sex** | **CVD risk (ASCVD score)** | **Viral load** | **CD4 count**  **(cells/mm^3^)** | **Memory Treg (cells/ml)** | **HDL (mg/dL)** | **LDL (mg/dL)** | **Total**  **cholesterol (mg/dL)** |  |
| --- | --- | --- | --- | --- | --- | --- | --- | --- | --- | --- |
| 1 | 62 | Male | 10.2% (Int/High) | <20 | 533 | 6915.25 | 48 | 115 | 202 |  |
| 2 | 65 | Female | 18.4% (Int/High) | <20 | 278 | 6904.45 | 44 | 110 | 187 |  |
| 3 | 43 | Female | 0.6% (Low/BL) | NA | 917 | 30449.20 | 48 | 118 | 186 |  |
| 4 | 64 | Male | 15.6% (Int/High) | NA | 805 | 3348.84 | 52 | 147 | 225 |  |
| 5 | 55 | Male | 6.6% (Low/BL) | <20 | 672 | 37278.82 | 50 | 54 | 113 |  |
| 6 | 58 | Female | 3.5% (Low/BL) | <20 | 927 | 27066.45 | 49 | 169 | 240 |  |
| 7 | 48 | Male | 3.9% (Low/BL) | <20 | 728 | 20666.39 | 58 | 68 | 163 |  |
| 8 | 51 | Male | 12.8% (Int/High) | <20 | 324 | 6262.84 | 35 | 128 | 182 |  |
| 9 | 50 | Male | 4.2% (Low/BL) | <20 | 533 | 23189.22 | 49 | 118 | 186 |  |
| 10 | 60 | Male | 8.6% (Int/High) | 93 | 763 | 7003.48 | 45 | 101 | 169 |  |

**Table S2. Characteristics of the HIV infected study participants that were chosen to isolate the HDL.**

**Supplementary Figure legends.**

**Figure S1. Isolated HDL from PWH.** Native SDS 4 – 15% gel analysis of MW: molecular weight standards and 1 - 10 HDL samples from PWH. H: Int/High CVD risk group and L: Low/BL CVD risk group

**Figure S2. Flow cytometry analysis and frequency of Treg subsets. A.** 130000 events from 43 samples were concatenated in the live gate. FOXP3+ T cells were pre-gating on singlets, live and CD3+CD4+ T cells. All the samples were normalized against a reference control using Cytonorm. Dimension reduction analysis and automated gating was performed by UMAP and FlowSOM. **B.** Frequency of Treg subsets in the concatenated file (43 samples) after Flowsom analysis.

**Figure S3. Treg absolute number inversely correlate with ASCVD.** Pearson correlation between **A.** Treg (FOXP3+), **B.** Memory Treg absolute numbers per mL and ASCVD in PWH with Low/BL or Int/High CVD risk. P and r values are displayed in the figure (* p<0.05 for significant differences).
