# Supplementary figures and images for: Impaired response of memory Treg to high density lipoproteins is associated with intermediate/high cardiovascular disease risk in persons with HIV
Source: Front Immunol. 2023 Mar 10;14:1146624. doi: 10.3389/fimmu.2023.1146624 (PMC10036595; doi:10.3389/fimmu.2023.1146624)

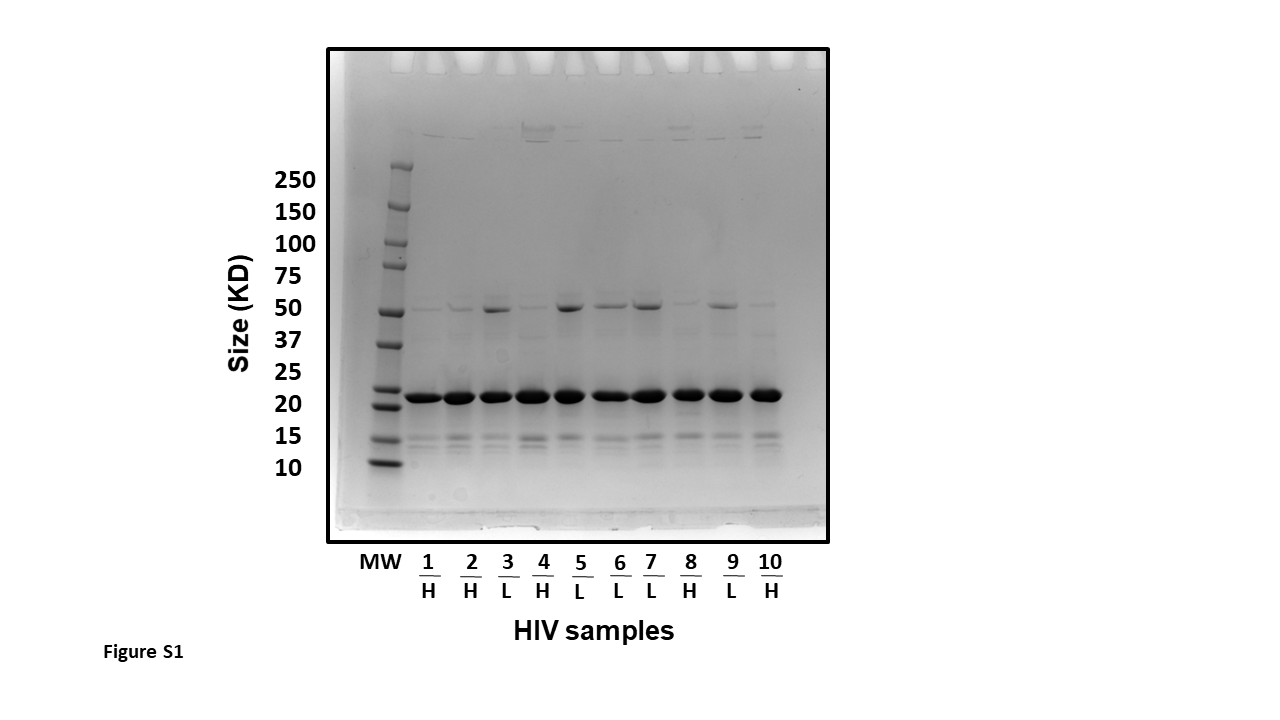

Supplement: Supplementary file 2 [file Image_1.tif]

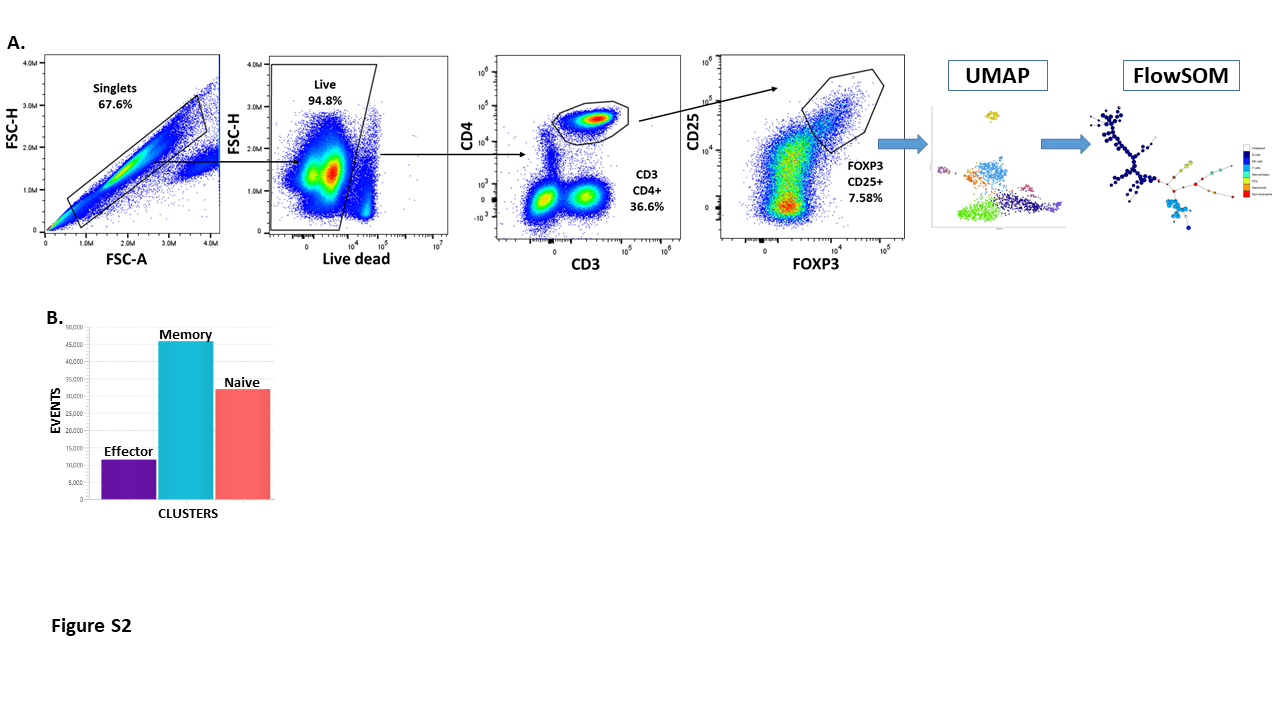

Supplement: Supplementary file 3 [file Image_2.tif]

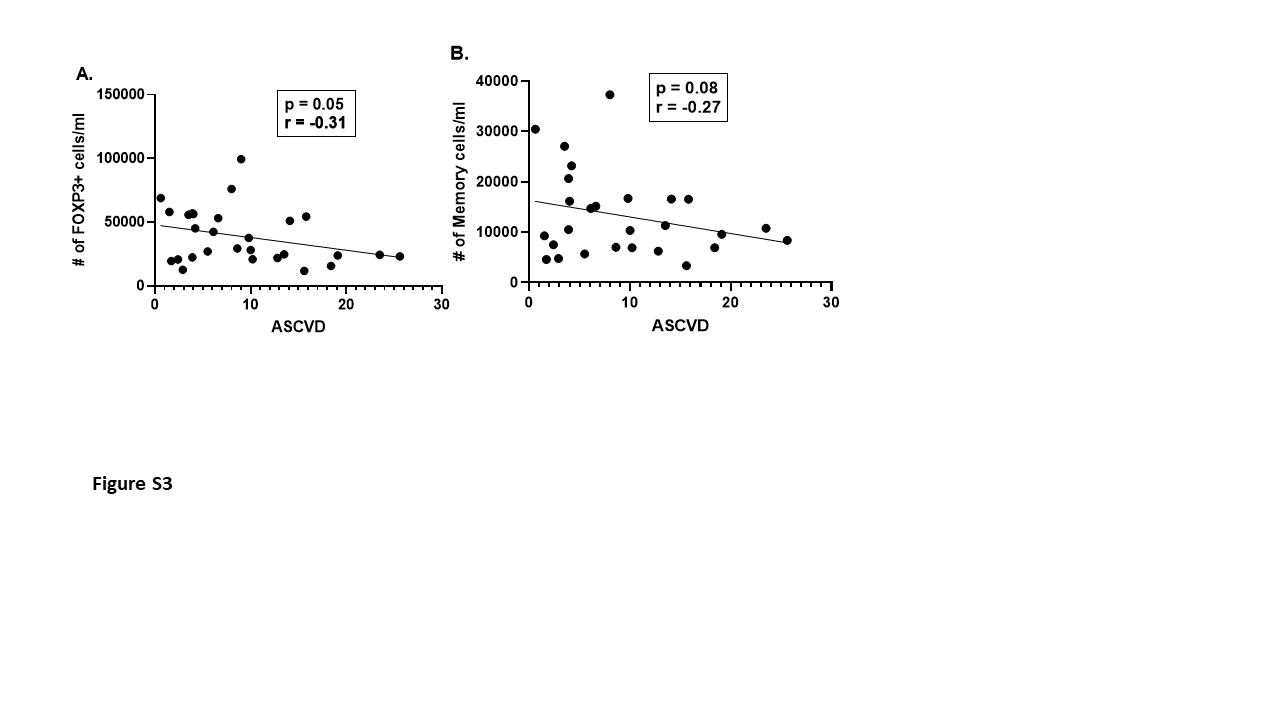

Supplement: Supplementary file 4 [file Image_3.tif]
